# Gene Expression Changes Associated with Resistance to Intravenous Corticosteroid Therapy in Children with Severe Ulcerative Colitis

**DOI:** 10.1371/journal.pone.0013085

**Published:** 2010-09-30

**Authors:** Boyko Kabakchiev, Dan Turner, Jeffrey Hyams, David Mack, Neal Leleiko, Wallace Crandall, James Markowitz, Anthony R. Otley, Wei Xu, Pingzhao Hu, Anne M. Griffiths, Mark S. Silverberg

**Affiliations:** 1 Mount Sinai Hospital, Toronto, Ontario, Canada; 2 The Hospital for Sick Children, Toronto, Ontario, Canada; 3 Izaak Walton Killam Hospital, Halifax, Nova Scotia, Canada; 4 Children's Hospital of Eastern Ontario, Ottawa, Ontario, Canada; 5 Connecticut Children's Medical Center, Hartford, Connecticut, United States of America; 6 Schneider's Children's Hospital, New Hyde Park, New York, United States of America; 7 Brown University, Providence, Rhode Island, United States of America; 8 Columbus Children's Hospital, Columbus, Ohio, United States of America; 9 Shaare Zedek Medical Center, Jerusalem, Israel; 10 University of Toronto, Toronto, Ontario, Canada; Emory University, United States of America

## Abstract

**Background and Aims:**

Microarray analysis of RNA expression allows gross examination of pathways operative in inflammation. We aimed to determine whether genes expressed in whole blood early following initiation of intravenous corticosteroid treatment can be associated with response.

**Methods:**

From a prospectively accrued cohort of 128 pediatric patients hospitalized for intravenous corticosteroid treatment of severe UC, we selected for analysis 20 corticosteroid responsive (hospital discharge or PUCAI ≤45 by day 5) and 20 corticosteroid resistant patients (need for second line medical therapy or colectomy, or PUCAI >45 by day 5). Total RNA was extracted from blood samples collected on day 3 of intravenous corticosteroid therapy. The eluted transcriptomes were quantified on Affymetrix Human Gene 1.0 ST arrays. The data was analysed by the local-pooled error method for discovery of differential gene expression and false discovery rate correction was applied to adjust for multiple comparisons.

**Results:**

A total of 41 genes differentially expressed between responders and non-responders were detected with statistical significance. Two of these genes, *CEACAM1* and *MMP8*, possibly inhibited by methylprednisolone through *IL8*, were both found to be over-expressed in non-responsive patients. *ABCC4* (*MRP4*) as a member of the multi-drug resistance superfamily was a novel candidate gene for corticosteroid resistance. The expression pattern of a cluster of 10 genes selected from the 41 significant hits were able to classify the patients with 80% sensitivity and 80% specificity.

**Conclusions:**

Elevated expression of several genes involved in inflammatory pathways was associated with resistance to intravenous corticosteroid therapy early in the course of treatment. Gene expression profiles may be useful to classify resistance to intravenous corticosteroids in children with severe UC and assist with clinical management decisions.

## Introduction

Although corticosteroids remain a mainstay of therapy for UC, a meta-regression of cohort studies in acute severe ulcerative colitis (UC) showed that 29% of patients fail corticosteroid therapy and require escalation of medical management or colectomy [1]. Evidence suggests that the dose of the administered corticosteroid and its bioavailability do not account for response or failure to therapy [1], [2]. Although some case series suggest that prolongation of corticosteroid therapy is effective in achieving remission in some refractory cases [3], this approach is associated with increased toxicity, discomfort, and cost. Several studies have attempted to identify predictors of corticosteroid response and allow for escalation of medical therapy or colectomy early during admission [1]. In adults, number of daily stools and C-reactive protein have good predictive ability on the third day of treatment [4], [5]. Albumin and bowel luminal width have been also associated with response to corticosteroid therapy [6]. In children, a predictive rule based on the Pediatric UC Activity Index (PUCAI) at days three and five of corticosteroid therapy has been shown to be superior to the adult scores [7]. A PUCAI value greater than 70 points should prompt initiation of second line therapy as was recently validated in a prospective cohort of children with severe UC, yielding positive predictive value (PPV) of 100% and negative predictive value (NPV) of 79% [8]. Although fecal calprotectin and pyruvate kinase have a fair predictive role, they do not add significantly to the clinical PUCAI score [9].

The expression of various proteins and genetic sequence alterations may contribute to corticosteroid resistance in asthma, rheumatic disease, and inflammatory bowel disease [10], [11], [12], [13]. For example, high expression levels of Multi Drug Resistance-1 (MDR-1) were found in UC patients who required colectomy [14]. MDR-1 may be involved in corticosteroid resistance by transporting the drug out across the cell membrane. Additionally, *in vitro* corticosteroid resistance of T-cells obtained from corticosteroid refractory UC patients no longer showed similar findings 3-months after discharge [15]. No differences in glucocorticoid receptor expression were observed in leukocytes obtained from previously corticosteroid responsive and resistant UC patients currently in remission [16].

RNA microarrays on 6 asthma patients revealed 9 genes, primarily involved in macrophage activation, to be differentially expressed between responders and non-responders to corticosteroids [17]. A different study by Hakonarson and colleagues identified over 900 transcripts which were differentially regulated between corticosteroid responsive and non-responsive asthma patients [18]. 15 of these transcripts could separate responders from non-responders with 84% accuracy [19]. No similar studies exist in UC. The aim of this prospective, multicenter study was to compare gene expression among children who responded to or failed intravenous corticosteroid therapy in acute, severe UC.

## Methods

### Study design

The evaluated patient population was from a nested case-control study of the *outcome of intravenous steroid therapy in ulcerative colitis individuals* (OSCI) study [20]. The OSCI study was a multicenter, prospective cohort study involving children, 2–18 years of age, hospitalized for intravenous corticosteroid therapy for acute UC. A diagnosis of UC was established by the presence of accepted clinical, radiologic, endoscopic and histological criteria [21]. The research ethics boards of the Hospital for Sick Children, Mount Sinai Hospital, Izaak Walton Killam Hospital, Children's Hospital of Eastern Ontario, and the institutional review boards of Connecticut Children's Medical Center, Schneider's Children's Hospital, the Children's Hospital of Philadelphia, Columbus Children's Hospital, and the Hasbro Children's Hospital approved this study. Informed, written consent and age-appropriate assent were obtained from participants and their caregiver, according to the local policy.

Pre-defined clinical, laboratory and radiographic data were collected on standardized case report forms at admission, on Day 3 and Day 5 of corticosteroid treatment, upon introduction of second line medical therapy (infliximab or calcineurin inhibitors) or colectomy (if applicable), and at hospital discharge. Disease activity was measured at each visit by the PUCAI [22] which is a non-invasive, 6-item index, ranging from 0 to 85, intended to measure disease activity in children with UC. This index was previously developed and validated by some of the authors using prospective cohorts and combined mathematical and judgmental strategies [7], [23], [24], [25]. As part of the OSCI study, in addition to clinical data, blood was collected for RNA extraction from all patients on Day 3 of corticosteroid treatment.

### Patient selection

The OSCI cohort consisted of 128 children and adolescents hospitalized for intravenous corticosteroid treatment of acute severe ulcerative colitis. Of these, 20 corticosteroid-responsive patients and 20 corticosteroid-refractory patients were selected for analysis of mRNA expression. All selected patients had been treated with methylprednisolone. Two batches of 20 patients, each composed of 10 non-responders and 10 responders, underwent microarray analysis (Table 1). Selection of subjects among the eligible non-responders (see below) was random for each batch. Responders of similar age and matching gender were selected in order to minimize potential confounding effects. To avoid selection bias, the inclusion of patients in the two groups was performed before the RNA assay was carried out and thus investigators were blinded to the expression results. Response was defined as no requirement for second line medical intervention or surgery by discharge and a PUCAI score less than or equal to 45 points by day 5 of therapy. Conversely, patients were categorized as non-responsive if they required second line medical therapy or surgery, or presented with a PUCAI score greater than 45 on day 5.

**Table 1:** Basic characteristics of included patients.

		Response to IVCS		
		Yes	No	p-value
**Batch 1**	N	10	10	-
	Male/Female	5/5	5/5	1.0000
	Age	14.4±2.2	14.6±2.2	0.852
	Disease Duration (mon.)	9±19	12±17	0.7372
	PUCAI Day 1	77±9	75±13	0.6986
	PUCAI Day 3	44±23	71±10	0.0033
	PUCAI Day 5	36±8	67±13	0.0012
**Batch 2**	N	10	10	-
	Male/Female	7/3	7/3	1.0000
	Age	12.7±2.6	14.6±2.4	0.1134
	Disease Duration (mon.)	18±39	20±35	0.8825
	PUCAI Day 1	66±11	73±8	0.1524
	PUCAI Day 3	34±13	61±14	0.0003
	PUCAI Day 5	19±11	54±16	0.0014
**Combined**	N	20	20	-
	Male/Female	12/8	12/8	1.0000
	Age	13.6±2.5	14.6±2.3	0.1831
	Disease Duration (mon.)	14±30	16±28	0.7778
	PUCAI Day 1	71±12	74±11	0.5217
	PUCAI Day 3	39±19	66±13	<0.0001
	PUCAI Day 5	27±13	61±16	<0.0001

N - Number of patients.

IVCS - Intravenous corticosteroids.

### Blood sample handling and RNA extraction

Blood samples were collected on Day 3 of intravenous corticosteroid therapy in *PAXgene* tubes (*PreAnalytiX*, *QIAGEN*) and stored at −80°C within 24 hours of collection for a period of less than 1 year. Total RNA was extracted with *PAXgene Blood RNA Kits* (*QIAGEN*) and the obtained elutes were stored at −80°C. The integrity of all RNA elutes was assessed with Bioanalyzer 2100 (*Agilent Technologies*) and only samples with RNA Integrity Number (RIN) greater than 5.5 were used for further analysis [26].

### Microarray analysis

Total RNA samples were hybridized to GeneChip Human Gene 1.0 ST Arrays (*Affymetrix*), with whole-transcript coverage of 28,869 genes and open reading frames (ORFs). GeneChip Whole Transcript (WT) Sense Target Labeling Assays with included quality control GeneChip Hybridization Control Kits (*Affymetrix*) were used for sample preparation. The chips were scanned and raw expression values were obtained with GeneChip Scanner 3000 (*Affymetrix*). (NCBI Gene Expression Omnibus series record - GSE21231).

### RT-PCR

The reliability of the microarray measurements was assessed through reverse-transcription real-time polymerase chain reaction (RT-PCR) measurements of the relative messenger RNA levels of 7 genes. These were selected based on their apparent importance to IBD disease mechanism. 6 pairs of specific primers were used to amplify exonic sequences in *OLFM4*, *MMP8*, *BPI*, *HP*, *CD177*, *DEFA1* and *DEFA3*. (Table S1) Due to the high homology between the sequences of *DEFA1* and *DEFA3* (99%), GeneChip Human Gene 1.0 ST Array probes measured the combined expression of the messenger RNA molecules. Likewise, RT-PCR primers were chosen to amplify a common segment between the two genes. The hypoxanthine phosphoribosyltransferase 1 (*HPRT1*) gene was used as an internal RT-PCR control. 300–600 ηg of total RNA were used with iScrip cDNA Synthesis Kits (*Bio-Rad*) to obtain complementary DNA. RT-PCR was performed with SYBR Green Supermix With ROX (*Bio-Rad*) and 15–30 ηg of template in a total reaction volume of 25 µL on 96-well plates.

### Data analysis

Probesets lacking annotation information were removed from further analysis. Raw data were background corrected, log transformed, and quantile normalized using a robust multi-array average (RMA) algorithm [27]. Within each batch, 21,176 genes and ORFs were correlated with a binary intravenous-corticosteroid therapy response variable across all samples using the local-pooled error (LPE) method for detection of significance [28]. Gene variance was estimated by pooling variance estimates of genes with similar expression from biological replicas across the response groups. Raw p-values were adjusted for multiple comparisons by the step-up FDR method [29]. A Monte Carlo simulation was used to estimate the expected overlap between the results from the two batches [30]. Only the most differentially expressed genes in each batch were considered for this analysis – 1059 (5% of total) genes with lowest p-values. Pearson's chi-squared test was used to compare the expected to the observed proportion of overlap. All 40 samples were first normalized separately, then scaled using the R function “scale” (http://www.r-project.org/), and finally pooled together into a single LPE analysis while controlling for batch effects.

Pharmacogenomic and gene-gene interaction data mining was accomplished via the Search Tool for Interactions of Chemicals (STITCH) [31]. Various aspects of molecular interaction were considered, including: activation, inhibition, binding, phenotypic similarity, catalysis, and co-expression.

The predictive value of genes correlated significantly with corticosteroid resistance was assessed by prediction analysis for microarrays (PAM). This approach utilizes the shrunken centroid method to identify genes which best characterize each response group [32]. The procedure was carried out in a 10-fold cross-validation fashion whereby the complete sample set was randomly divided into 10 subsets of equal size. Each of the 10 subsets was consecutively used for validating a classifier which was trained on the remaining 9 subsets. A classification score for each sample was determined based on the distance to the nearest shrunken centroid. The performance of the classifier was then averaged over the 10 validation events (folds). This cross-validation approach is very robust and preferred for analyzing fewer than 50 samples due to its high data utilization efficiency [33].

## Results

After screening the entire OSCI study cohort, 40 patients were included according to the predefined criteria as described in Table 1. Separate LPE analyses of each batch of samples produced very similar results. When ranked by p-value, an overlap of 341 genes was found between the 5% (1059 genes) of genes with lowest p-values in both batches. (Figure S1) In other words, the observed overlap between the results from the two separate analyses was 32.2%. A Monte Carlo simulation indicated that the mean expected overlap, assuming no correlation between the two batches, was 52.963±0.007 genes with a median of 53 genes (5%). A chi-squared test showed that there was a very significant difference between the observed and expected proportion of overlap (p = 4.18×10^−57^).

After correction for multiple testing, 41 genes expressed differentially between responders and non-responders to therapy were detected with statistical significance in the pooled analysis. (Table 2) Some of the most significant genes were Olfactomedin 4 (*OLFM4*), Matrix Metallopeptidase 8 (*MMP8*), RAP1 GTPase Activating Protein (*RAP1GAP*), 6-phosphofructo-2-kinase (*PFKFB2*), Bactericidal Permeability-increasing Protein (*BPI*), and N-terminal EF-hand Calcium Binding Protein 1 (*NECAB1*). The expression of all significant genes was elevated in patients who had no response to the administered therapy.

**Table 2:** List of genes significantly associated with response to intravenous corticosteroid therapy.

FDR Adjusted P-value	Fold Change	Gene Symbol	Description	Immune System Associated
<0.0001	1.8	**OLFM4**	Olfactomedin 4	Yes
<0.0001	1.9	**MMP8**	Matrix Metallopeptidase 8 (Neutrophil Collagenase)	Yes
<0.0001	1.6	**RAP1GAP**	RAP1 GTPase Activating Protein	Yes
<0.0001	1.3	**PFKFB2**	6-phosphofructo-2-kinase	-
<0.0001	1.3	**BPI**	Bactericidal Permeability-increasing Protein	Yes
<0.0001	1.7	**NECAB1**	N-terminal EF-hand Calcium Binding Protein 1	-
<0.0001	2.5	**CLC**	Charcot-Leyden Crystal Protein	Yes
<0.0001	1.8	**TSTA3**	Tissue Specific Transplantation Antigen P35B	Yes
<0.0001	1.8	**GPR84**	G Protein-coupled Receptor 84	-
<0.0001	1.3	**RPS26P2**	Ribosomal Protein S26 Pseudogene 2	-
<0.0001	1.6	**HP**	Haptoglobin	Yes
0.0001	1.2	**MS4A3**	Membrane-spanning 4-domains	Yes
0.0001	1.4	**SERPINB10**	Serpin Peptidase Inhibitor	Yes
0.0001	1.4	**TDRD9**	Tudor Domain Containing 9	-
0.0003	1.3	**LCN2**	Lipocalin 2	Yes
0.0012	2.0	**CD177**	CD177 Molecule	Yes
0.0012	1.3	**RPS26P15**	Ribosomal Protein S26 Pseudogene 15	-
0.0013	1.5	**GMPR**	Guanosine Monophosphate Reductase	-
0.0017	1.4	**CLIC2**	Chloride Intracellular Channel 2	-
0.0027	1.5	**PROS1**	Protein S (Alpha)	Yes
0.0063	1.6	**TCN1**	Transcobalamin I (Vitamin B12 Binding Protein)	Yes
0.0072	1.5	**CEACAM1**	Carcinoembryonic Antigen-related Cell Adhesion Molecule 1	Yes
0.0073	1.5	**HEPACAM2**	HEPACAM family member 2	Yes
0.0073	1.5	**ATP9A**	ATPase	-
0.0082	1.2	**RPS26L**	40S Ribosomal Protein S26-like	-
0.0128	1.2	**DEFA1/DEFA3**	Defensin	Yes
0.0129	1.3	**MBNL3**	Muscleblind-like 3 (Drosophila)	-
0.0155	1.4	**PPBP**	Pro-platelet Basic Protein	Yes
0.0221	1.6	**ITGB3**	Integrin	Yes
0.0244	1.2	**CLEC4C**	C-type Lectin Domain Family 4	Yes
0.0257	1.5	**GYPC**	Glycophorin C (Gerbich blood group)	-
0.0257	1.3	**CA1**	Carbonic Anhydrase I	-
0.0284	1.6	**ENTPD7**	Ectonucleoside Triphosphate Diphosphohydrolase 7	-
0.0284	1.3	**VSTM1**	V-set and Transmembrane Domain Containing 1	-
0.0361	1.2	**SUCNR1**	Succinate Receptor 1	-
0.0445	1.6	**ELOVL7**	ELOVL Family Member 7	-
0.0445	1.2	**PSTPIP2**	Proline-serine-threonine Phosphatase Interacting Protein 2	-
0.0459	2.2	**RNF182**	Ring Finger Protein 182	-
0.0459	1.3	**ABCC4**	ATP-binding Cassette	Yes
0.0459	1.8	**GPR146**	G Protein-coupled Receptor 146	-

Genes with statistically significant association to intravenous corticosteroid therapy response after correction for multiple testing (FDR-adjusted p-values<0.05). The average fold increase in expression in non-responsive compared to responsive patients, official gene symbol, a short description, and prior evidence of immune pathway involvement are listed for each gene.

RT-PCR was used to confirm the relative expression obtained from the microarray experiment. The transcripts of *OLFM4*, *MMP8*, *BPI*, *HP*, *CD177*, *DEFA1* and *DEFA3* were successfully measured in real-time using the primers listed in Table S1. We demonstrated that the RT-PCR and microarray expression values were highly correlated confirming the validity of the microarray results. (Figure S2)

**Figure 1:**
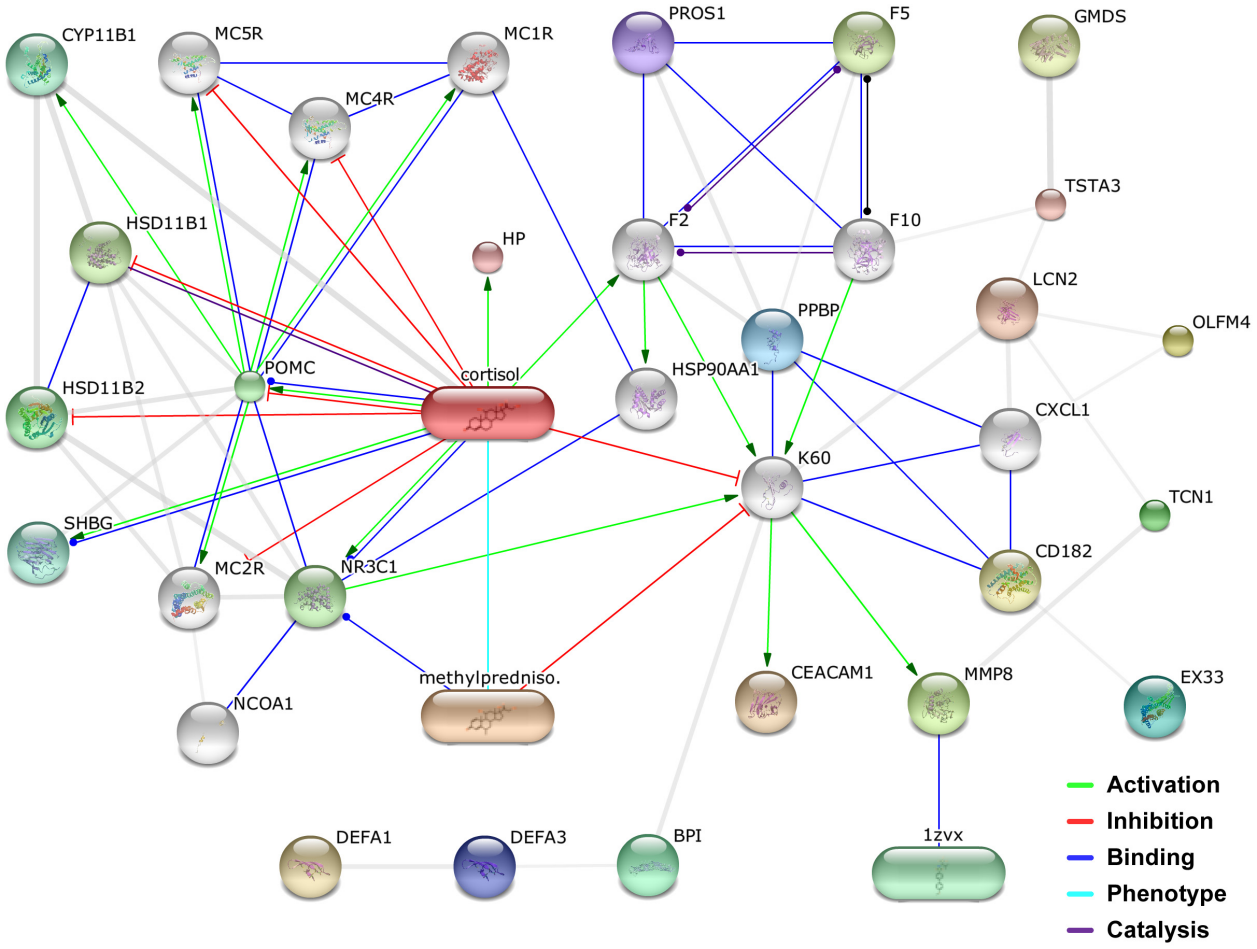
Immediate interactome of methylprednisolone. Point and block arrows indicate activation or inhibition, respectively. Grey lines represent interactions of undetermined nature, while coloured lines indicate evidence for specific interactions.

Intriguing information on the interconnectivity of some of the 41 significant genes was obtained through network analysis. A diagrammatic representation of the immediate interactome of methylprednisolone indicated an inhibitory action on *K60*, also known as *IL8* (Figure 1) [34]. In turn, *IL8* is a known inducer of *CEACAM1* and *MMP8* [35], [36], [37], and it also interacts with *BPI*, *LCN2* and *PPBP* [36], [38], [39], [40]. All of these partners of *IL8* were expressed significantly higher in our patients who had no response to intravenous corticosteroid therapy. A study by Matsuda *et al.* showed that *IL8* mRNA levels are high during active disease and low during quiescent disease in UC patients [41]. However, 4% elevation of *IL8* expression levels in our cohort of corticosteroid non-responsive individuals was not statistically significant (FDR p = 1).

Finally, the predictive value of the total and various subsets of the 41 differentially-expressed genes was assessed. (Table 3) Using all 41 genes, it was possible to classify the 40 tested samples as belonging to either corticosteroid resistant or responsive patients with 80% sensitivity and 70% specificity. Subgroups including the top 40, 35, 30, 25, 20, 15, 10, or 5 of differentially expressed genes performed similarly as classifiers. While the specificity and sensitivity decreased with the number of tested genes, neither value dropped below 60%. Interestingly, the 10 best classifiers of corticosteroid resistance – *RAP1GAP*, *CD177*, *ELOVL7*, *CLIC2*, *TSTA3*, *HP*, *MMP8*, *NECAB1*, *PROS1* and *ITGB3* – were 80% specific and 80% sensitive. (Table 4) In a completely unbiased PAM analysis, 9 out of these 10 classifier genes remained within the 46 best classifiers out of all 21, 176 tested genes. In this analysis, the most accurate classification was achieved by the use of 662 genes (data not shown) with specificity and sensitivity of 75%.

**Table 3:** Class prediction analysis for microarrays using nearest shrunken centroid method.

Most Significant Genes (N)	Specificity	Sensitivity	Accuracy
41	80%	70%	75%
40	75%	75%	75%
35	80%	70%	75%
30	70%	70%	70%
25	75%	65%	70%
20	75%	70%	73%
15	70%	60%	65%
10	75%	70%	73%
5	65%	65%	65%

Assessment of the specificity, sensitivity and accuracy for the N most significant genes (where N equals 41 through 5 in decreasing increments of 5).

Accuracy % = 100 * ( True Positives+True Negatives )/Total.

**Table 4:** Specificity, sensitivity and accuracy of the 10 best indicators of corticosteroid resistance.

10 Best Indicators of Corticosteroid Resistance	Specificity	Sensitivity	Accuracy
RAP1GAP, CD177, ELOVL7, CLIC2, TSTA3, HP, MMP8, NECAB1, PROS1, ITGB3	80%	80%	80%

## Discussion

Whole-genome expression analysis of patients receiving intravenous corticosteroid revealed 41 genes with expression levels significantly associated with resistance to the therapy. A substantial proportion of these genes are known to be involved in the inflammatory response. Furthermore, mining molecular interaction databases provided a basis for interpreting how these expression results fit the paradigm of corticosteroid resistance. Of potential clinical relevance was a set of 10 genes which classified the treated patients with 80% sensitivity and specificity. Conceivably, measurement of the expression levels of these genes could be developed into a practical tool to help identify individuals who are likely to fail such intravenous corticosteroid therapy and improve their medication course.

While gene expression changes that occur during treatment may help elucidate the mechanism of corticosteroid resistance, it is essential to further investigate the various molecular interactions alluded to in this study. All genes found to be good classifiers of corticosteroid resistance must necessarily be validated in a large, prospective study. Gene expression changes observed on the third day of therapy are likely to highlight pathways relevant to corticosteroid metabolism and may be temporal. Conversely, sampling patients prior to the initiation of therapy would establish whether the discovered pathways are intrinsic to the individual's disease behaviour.

Network analysis revealed that IL-8 function provides a possible explanation for the observed overexpression of *CEACAM1* and *MMP8* in non-responsive patients [35], [36], [37]. Corticosteroid resistance could result in reduced inhibition of *IL8* by methylprednisolone and thus affect its downstream interactions [34]. *IL8* expression was not significantly different between responders and non-responders in this study despite a previous report of elevated *IL8* mRNA levels in active UC [41]. However, methylprednisolone appears to inhibit IL-8 by preventing its release rather than by affecting its gene expression [34]. Indeed, this claim is partially supported by evidence that IL-8 can be stored in Weibel-Palade bodies for rapid release [42] and its protein levels could remain briefly stable after changes in its gene expression.

A number of the significant genes identified in this study are of specific interest due to their prior association with IBD or pathways implicated in IBD. Olfactomedin 4 (*OLFM4*) is a gene encoding a member of the olfactomedin-related protein family. Selective over-expression of *OLFM4* has been reported in inflamed colonic crypt epithelium in ulcerative colitis patients [43]. The exact function of the protein, however, is not known. A study by Zhang *et al.* indicates that *OLFM4* is an anti-apoptotic factor which attenuates the ability of *GRIM19* to facilitate retinoic acid-IFN-β-mediated apoptosis and the expression of apoptotic genes [44]. Barnich *et al.* report that *GRIM19* interacts with nucleotide oligomerization domain 2 (*NOD2*) and is required for the activation of *NF-κB* [45]. Interestingly, Liu *et al.* recently demonstrated that *OLFM4* down-regulates the innate immune response by influencing NOD1 and NOD2 mediated NF-κB activation in a mouse model of *Helicobacter pylori* infection [46]. Other reports implicate *OLFM4* expression in tumour growth and, more specifically, in colon cancer [47], [48]. *OLFM4* has also been shown to bind cell-surface lectins and cadherin [49].

Matrix metalloproteinase 8 (*MMP8*), a collagenase secreted by neutrophils, was also expressed more highly in patients who were not responding to intravenous corticosteroids. This proteinase can degrade type I, II and III collagen and thus affect the extracellular matrix. A study by Schaaf et al. shows elevated activity of MMP8 in hospitalized patients in the presence of bacteria compared to controls [50]. Alpha defensin 1 (*DEFA1*) and 3 (*DEFA3*) as well as bactericidal/permeability-increasing protein (*BPI*) are peptides secreted by neutrophils in response to bacterial antigens. Elevated levels of all three proteins have been associated with inflammatory activity in rheumatoid arthritis [51]. *DEFA1* and *DEFA3* have also been implicated in lung epithelial wound repair [52].

*HP* encodes both the alpha and beta chains of the haptoglobin tetramer, a protein responsible for the clearance of free plasma haemoglobin and a mediator of the inflammatory response. *HP* is an inducer of IL-6 and plays a role in the balance of Th1 and Th2 cell populations [53], [54]. Polymorphisms in this gene have been associated with Crohn's disease, disease behaviour and extraintestinal manifestations [55]. *CD177* is a glycoprotein selectively expressed by neutrophils and found on their surface. Although its exact function is not known, it has been identified as a binding partner of platelet endothelial cell adhesion molecule-1 (*PECAM-1*) and it may play a crucial role in the extravasation of neutrophils into tissues [56]. *CD177* expression is increased in individuals with severe bacterial infections and polycythaemia vera, but not rheumatoid arthritis [57]. In our study, expression of *CD177* was increased two-fold in patients who did not respond to intravenous corticosteroid therapy compared to those who did well.

ATP-binding cassette, sub-family C, member 4 (*ABCC4*) is a gene that may directly influence corticosteroid response. *ABCC4* is also known as multi-drug resistance protein 4 (*MRP4*) and is part of the same superfamily as *MDR1*. ABCC4 is an ATP-dependent transporter and it has been associated with resistance to multiple drugs. More specifically, it has been shown to actively transport prostaglandins, methotrexate and steroid- and bile acid-conjugates [58], [59], [60].

The results of this study demonstrate that significant gene expression differences exist between patients who respond to intravenous corticosteroid therapy and those who are resistant. It also validates the use of RNA expression analysis as a useful approach toward improving the understanding of disease processes and response to medical therapy. Clinically meaningful tools may also be developed that would allow for the early stratification of patients into prognostic categories that would individualize care approaches.

### Web Resources

NCBI Gene Expression Omnibus (GEO) - http://www.ncbi.nlm.nih.gov/geo/

R function “scale” - http://www.r-project.org/

Search Tool for Interactions of Chemicals - http://stitch.embl.de/

## Supporting Information

Figure S1 Graphical representation of the analysis used to compare the results from batch 1 and batch2. The expected overlap under the null hypothesis was obtained by a Monte Carlo simulation.
(1.10 MB DOC)

Figure S2 Scatterplots illustrate data for genes in each batch as well as a combined analysis, including R2 values.
(3.40 MB DOC)

Table S1 Forward and reverse primers and corresponding length of RT-PCR products are listed for each of the 7 amplified genes.
(0.03 MB DOC)
