# Supplementary material for: Gene Expression Changes Associated with Resistance to Intravenous Corticosteroid Therapy in Children with Severe Ulcerative Colitis
Source: PLoS One. 2010 Sep 30;5(9):e13085. doi: 10.1371/journal.pone.0013085 (PMC2948001; doi:10.1371/journal.pone.0013085)
Supplement: Figure S1 — Graphical representation of the analysis used to compare the results from batch 1 and batch2. The expected overlap under the null hypothesis was obtained by a Monte Carlo simulation. (1.10 MB DOC) [file pone.0013085.s001.doc]

**Figure S1. Batch comparison.** Graphical representation of the analysis used to compare the results from batch 1 and batch2. The expected overlap under the null hypothesis was obtained by a Monte Carlo simulation.

**
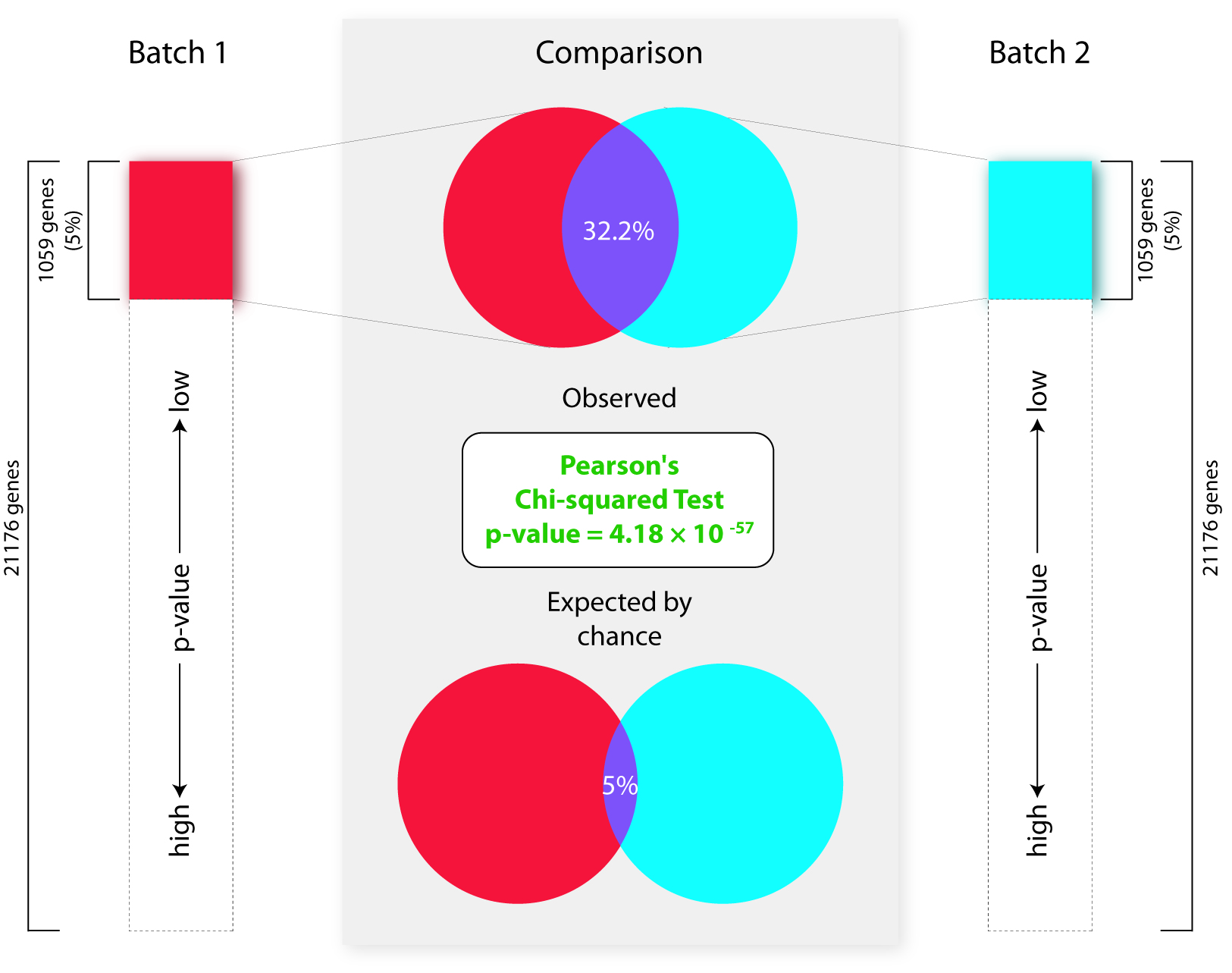
**
