# Supplementary material for: Gene Expression Changes Associated with Resistance to Intravenous Corticosteroid Therapy in Children with Severe Ulcerative Colitis
Source: PLoS One. 2010 Sep 30;5(9):e13085. doi: 10.1371/journal.pone.0013085 (PMC2948001; doi:10.1371/journal.pone.0013085)
Supplement: Figure S2 — Scatterplots illustrate data for genes in each batch as well as a combined analysis, including R2 values. (3.40 MB DOC) [file pone.0013085.s002.doc]

**Figure S2. Comparison of relative microarray and RT-PCR expression.** Scatterplots illustrate data for genes in each batch as well as a combined analysis, including R^2^ values.

**
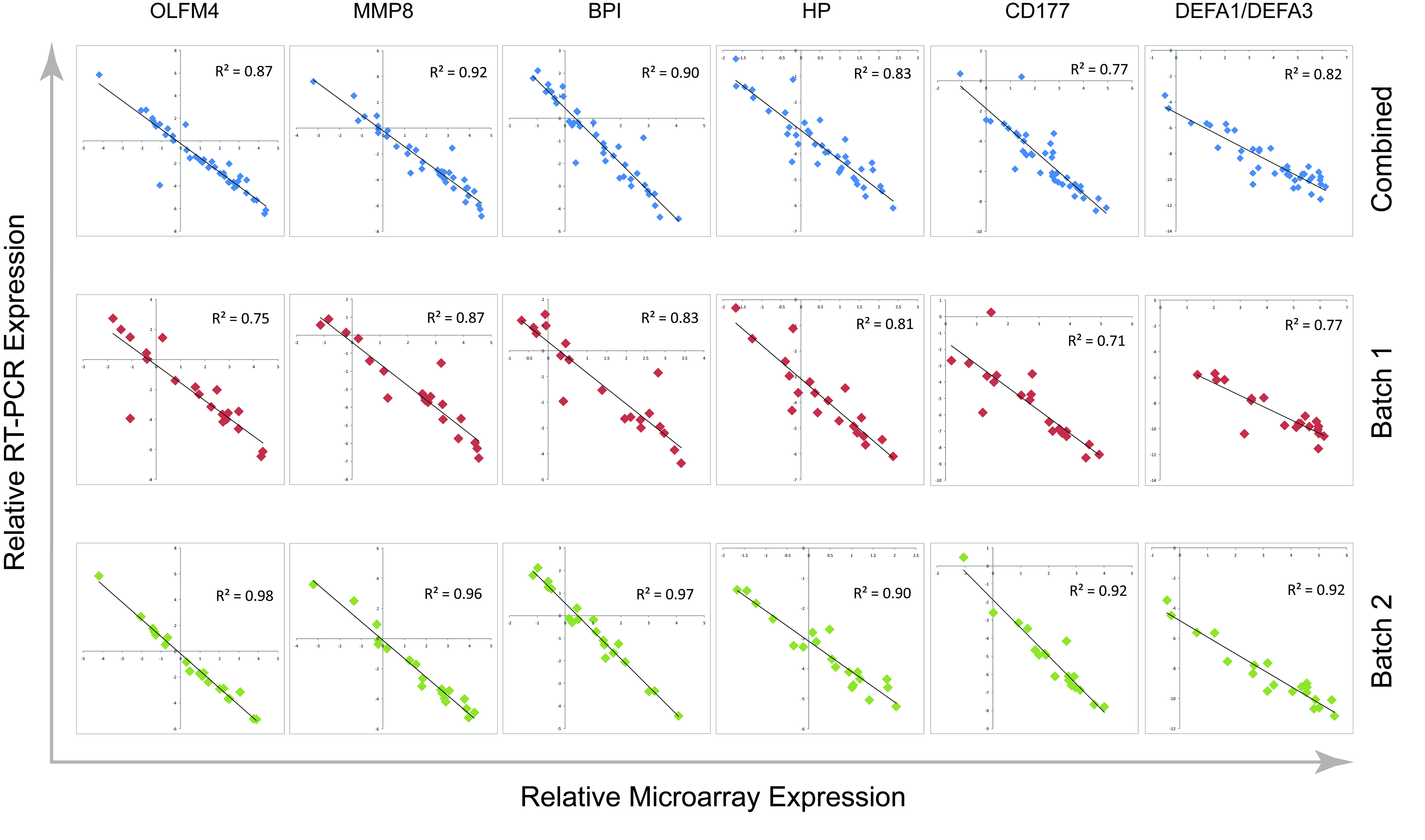
**
