# Supplementary material for: Gene Expression Changes Associated with Resistance to Intravenous Corticosteroid Therapy in Children with Severe Ulcerative Colitis
Source: PLoS One. 2010 Sep 30;5(9):e13085. doi: 10.1371/journal.pone.0013085 (PMC2948001; doi:10.1371/journal.pone.0013085)
Supplement: Table S1 — Forward and reverse primers and corresponding length of RT-PCR products are listed for each of the 7 amplified genes. (0.03 MB DOC) [file pone.0013085.s003.doc]

**Table S1. Forward and reverse primers and corresponding length of RT-PCR products are listed for each of the 7 amplified genes.**

| **Gene** | **Forward Primer** | **Reverse Primer** | **Product Length**  **(bp)** |
| --- | --- | --- | --- |
| **OLFM4** | CAGCCCCGGCTTCAGCTCTT | ACTGGCAGGTCCCACGGTCA | 164 |
| **MMP8** | TGGGGCTCGCTCACTCCTCTG | GGGGTCACAGGGTTTGGGTGT | 176 |
| **BPI** | ACCCCGCCACACCTGTCTGT | CTGTTGGACTCGGCGCTGACC | 158 |
| **HP** | TTATGCAGAAGTAGGGCGTG | CGGTGTCTTCTTTTCGGGGA | 160 |
| **CD177** | TCAGGCTCAGGGGAGGAGGC | GGTGGTCCCCCGATGACAGGT | 161 |
| **DEFA1 / DEFA3** | ACCAGCGTGCATTGCAGGAGA | AGGAAAGGAAATTGAGCAGAAGGT | 169 |
| **HPRT1** | AGCCCTGGCGTCGTGATTAGT | CCATCTCCTTCATCACATCTC | 160 |
